# Thyroxine transfer from cerebrospinal fluid into choroid plexus and brain is affected by brefeldin A, low sodium, BCH, and phloretin, in ventriculo-cisternal perfused rabbits

**DOI:** 10.3389/fcell.2015.00060

**Published:** 2015-09-29

**Authors:** Kazem Zibara, Ali El-Zein, Wissam Joumaa, Mohammad El-Sayyad, Stefania Mondello, Nouhad Kassem

**Affiliations:** ^1^ER045, PRASE, Faculty of Sciences-I, Lebanese UniversityBeirut, Lebanon; ^2^Department of Biology, Faculty of Sciences-I, Lebanese UniversityBeirut, Lebanon; ^3^Physics Department, Faculty of Sciences, Lebanese UniversityBeirut, Lebanon; ^4^Department of Family Medicine, University of ToledoToledo, OH, USA; ^5^Department of Neurosciences, University of MessinaMessina, Italy; ^6^Department of Pharmacology and Therapeutics, Institute of Pharmaceutical Science, King's College LondonLondon, UK

**Keywords:** thyroid hormone, transport, blood-CSF barrier, blood-brain barrier

## Abstract

**Background:** Thyroxine (T_4_) hormone is synthesized by the thyroid gland and then released into the systemic circulation where it binds to a number of proteins. Dysfunction in T_4_ transport mechanisms has been demonstrated in multiple central nervous system (CNS) diseases including Alzheimer's disease. In the presence of different compounds that inhibit potential T_4_ transport mechanisms, this study investigated the transfer of T_4_ from cerebrospinal fluid (CSF) into Choroid Plexus (CP) and other brain tissues. The compounds used were brefeldin A, low sodium artificial CSF (aCSF), BCH, phloretin, and taurocholate (TA).

**Methods:** Radiolabeled T_4_ (^125^I-T_4_) was perfused continuously into the CSF and was assessed in several brain compartments with reference molecule ^14^C-mannitol and blue dextran, using the *in vivo* ventriculo-cisternal perfusion (V-C) technique in the rabbit. The aCSF containing the drug of interest was infused after 1 h of perfusion. Drugs were applied independently to the aCSF after 1 h of control perfusion.

**Results:** Of interest, in presence of low sodium or BCH, the percentage recovery of ^125^I-T_4_, was increased compared to controls, with concomitant increase in T_4_ clearance. Conversely, brefeldin A, phloretin, and TA did not exert any significant effect on the recovery and clearance of ^125^I-T_4_ assessed in aCSF. On the other hand, the uptake of ^125^I-T_4_ into CP was raised by 18 fold compared to controls in the presence of brefeldin A. In addition, low sodium, BCH, or phloretin alone, enhanced the uptake of ^125^I-T_4_ by almost 3-fold, whereas TA did not show any significant effect. Finally, the uptake and distribution of ^125^I-T_4_ into other brain regions including ependymal region (ER) and caudate putamen (CAP) were significantly higher than in controls.

**Conclusion:** Our study suggests the involvement of different mechanisms for the transfer of ^125^I-T_4_ from CSF into CP and other brain regions. This transfer may implicate sodium-dependent mechanisms, amino acid “L” system, or organic anion transporting polypeptide (OATP).

## Introduction

Thyroid hormone (TH) is essential for normal growth, development of the central nervous system (CNS) (Goncalves et al., 1990; Koibuchi, 2013), and neuronal regeneration in adults after traumatic brain injury (TBI) (Francon et al., 1989). Thyroxine hormone (T_4_) is synthesized by the thyroid gland and then released into the systemic circulation where it binds to a number of proteins such as albumin (ALB), transthyretin (TTR), and thyroid binding globulin (TBG) (Baehr et al., 2006). Hypothyroxinemia causes neurological deficit and mental retardation in the fetus (Wei et al., 2013). In order for T_4_ to reach the CNS, it must first cross the brain barriers, i.e., endothelial cells of the blood-brain barrier (BBB) and epithelial cells of the choroid plexus (CP) of blood-cerebrospinal fluid barrier (BCSF-B). Dysfunction in T_4_ transport mechanisms has been demonstrated in multiple diseases of the CNS. In fact, low levels of free T_4_ in the CSF have been demonstrated in patients with multiple sclerosis (Pieragostino et al., 2013) and Alzheimer's Disease (Johansson et al., 2013).

Despite the importance of studying the direction of T_4_ transport from blood into CNS, the underlying mechanisms responsible for its entry from CSF into CP and brain, has not been fully investigated. Few studies in mice revealed that T_4_ transport across BBB is primarily directed from brain to blood via carrier-mediated mechanism (Banks et al., 1985). However, T_4_ injected directly into the CSF compartment in the rat penetrated very poorly into the brain (Dratman et al., 1991; Blay et al., 1993). In the rabbit, our laboratory have demonstrated that T_4_ transport from CSF into CP's and other brain tissues is carrier-mediated and dependent on the presence of T_4_ binding protein TTR, synthesized by the CP's (Kassem et al., 2006). Moreover, we have also shown that in isolated perfused sheep CP, T_4_ transport is partially dependent on sodium (Kassem et al., 2002).

Previous research has concentrated on studying the transport direction of T_4_ from blood into the brain across the BBB suggesting a role for organic anion transporting polypeptides (OATP) such as OATP 1a4, 1c1, 2, 3, and 14, which are mainly localized at the blood side of the BBB (Abe et al., 1998; Gao and Meier, 2001; Mayerl et al., 2012; Do et al., 2013) and CP (Gao et al., 1999; Gao and Meier, 2001; Mayerl et al., 2012). On the other hand, others have demonstrated a role for OATP2 and multiple drug resistance 1 (MDR1) in the transport of ^125^I-T_4_ at the basolateral side of the isolated perfused sheep CPs (Preston and Segal, 1992). Furthermore, a potential role for P-glycoprotein (P-gp) and OATP 1 & 3 was found in T_4_ transport mechanism (Kassem et al., 2007). Finally, human OATP 1c1 has been reported to have a high specificity for T_4_ and is considered to play a critical role in its transport across the BBB (Jansen et al., 2005; van der Deure et al., 2008). However, monocarboxylate (MCT8) has been shown to transport T_3_ and T_4_ THs (Grijota-Martínez et al., 2011).

The aim of this study is to investigate the effect of several cross-competing drugs on the transport of ^125^I-T_4_ from CSF compartment into CP and the brain, using *Ventriculo-Cisternal (V-C)* perfusion in the rabbit, and to potentially characterize putative transporters contributing to this process. The following drugs/conditions were used: brefeldin A (a blocker of intracellular secretory proteins such as TTR, Klausner et al., 1992), low sodium artificial CSF (aCSF), β-2-aminobicyclo-(2,2.1)-heptane-2-carboxylic acid (BCH) (an amino acid analog inhibitor of L-type transporters, Braun et al., 2011), phloretin (a sugar analog), and taurocholate (TA) (an OATP inhibitor).

## Materials and methods

### Animal handling

All procedures were conducted according to the Home Office legislation of the animal scientific procedures in compliance with the guidelines of the current animal legislation Act, 1986 UK. This study was approved by the Institute Animal Care and Utilization Committee of King's College London.

### Ventriculo-cisternal (V-C) perfusion

This method has been previously described (Davson and Segal, 1970; Kassem et al., 2006, 2007). Briefly, 1.5–4 Kg New Zealand white rabbits of either sex were anesthetized by the intravenous injection of a mixture of sodium Pentobarbital (10 mg/kg^−1^, Sagatal, Sigma, UK) and Medetomidine hydrochloride (0.5 mg/kg^−1^, Domitor, Sigma, UK). Bilateral perfusion of the ventricular system was carried out by placing two catheter inflow needles into each lateral ventricle (Kassem et al., 2006). The aCSF was infused into both ventricles by a pump (Harvard 22, UK) at a total rate of 60 μl/min, containing ^125^I-labeled T_4_ (0.37 MBq/40 ml), and the extracellular marker mannitol (0.148 MBq/40 ml, New England Nuclear, UK). Artificial CSF contained (in mmol/l): 153 Na^+^, 2.81 K^+^, 1.7 Mg^2+^, 2.81 Ca^2+^, 131 Cl^−^, 1.7 ${\text{SO}}_{4}^{2-}$, 1.48 ${\text{PO}}_{4}^{2-}$, 27.4 ${\text{HCO}}_{3}^{-}$, and 5.3 glucose. The solution was then gassed with 95% O_2_ and 5% CO_2_. Low sodium aCSF was used by replacing the NaCl and KCl in the perfusate with choline chloride. The sodium content in the aCSF was made up from ${\text{NaHCO}}_{3}^{-}$, which contained ~27 mM Na. Blue dextran, a large marker molecule of 2 × 10^6^ kDa M.wt. (Sigma, UK) was confined to the aCSF and used to determine the secretion rate (K_*f*_) of the newly formed CSF (Davson and Segal, 1970; Kassem et al., 2006). The outflow perfusate was collected continuously, every 10 min, from a single needle positioned in the cisterna magna. Perfusion time lasted for 2 h, in all experiments.

### Sampling of brain tissues and isotopes counting

This was performed as previously described (Kassem et al., 2006, 2007). Briefly, rabbits were sacrificed, their brains rapidly removed, and ventricles opened and flushed with 0.9% saline. The brain hemispheres were split through the midline and cut into series of coronal sections. The CP's were removed, and the ventricular ependymal region (ER) of the frontal cortex was dissected, as 1 mm thick layer (20 mg) from the area next to the midline (Preston and Segal, 1992; Preston et al., 2005). The ER was then gently peeled back from the rest of ventricular wall by a horizontal cut. The uptake of ^125^I-T_4_ into ER was compared to tissue samples designated the subependymal region (SER), located immediately below the dissected region (1 mm thick, 20 mg). The rest of the brain was dissected into hippocampus (HC) with or without ER (HC+ER or HC-ER), respectively. The caudate putamen (CAP) was also processed.

### Treatments

aCSF inflow and outflow samples (100 μl in triplicate), and tissue samples were dissolved in 0.5 ml Solusol (National diagnostic, UK). A total of 3.5 ml of scintillation liquid was added (Ultima Gold, Packard, UK) and samples were counted with LKB Wallac-1219 Beta liquid scintillation counter. Both ^125^I and ^14^C were expressed as disintegration per minute (dpm).

### Drugs

After control aCSF perfusion for 1 h, one of the following drugs was separately added to the aCSF for another 1 h: brefeldin A (5.0 μg/ml) (Lei et al., 2003), low sodium (~27 mM), BCH (5.0 mM) (Preston et al., 2005), phloretin (100 μM) (Deane and Segal, 1979), or taurocholate (1.0 mM) (Kitazawa et al., 2000). Drugs were dissolved directly in aCSF. The effect of each drug was compared to its own control experiment. The drugs were applied to separate animals. The control for each experimental animal was done before applying the specific drug, therefore; the effect of each investigated drug was animal specific. Results represents the average effect of each drug on *n* = 3–4 animals.

### Expression of results

#### Percent recovery of ^125^I-T_4_ in aCSF

As previously published (Bradbury and Davson, 1964; Kassem et al., 2006), the % of ^125^I-T_4_ remaining in outflow aCSF, in each 10 min sample, was calculated from: % recovery = (C_out_/C_in_) × 100, where the *C*_out_ = ^125^I-T_4_ dpm.ml^−1^, *C*_in_ = ^125^I-T_4_ dpm.ml^−1^ inflow aCSF. The steady-state ratio of ^125^I-T_4_ in the recovered aCSF was calculated from the mean value of % recovery derived from the last four samples of perfusion fluid.

#### Clearance of ^125^I-T_4_ from aCSF

The rate of ^125^I-T_4_ loss from the aCSF during V-C perfusion was expressed as clearance, defined as the volume (μl) of aCSF cleared of ^125^I-T_4_ per minute. The equation employed has previously been described (Kassem et al., 2006):

$$\begin{array}{l} \text{Clearance}\left(\mu \text{l}.\text{mi}{\text{n}}^{-1}\right)={\text{F}}_{\text{in}}\left({\text{C}}_{\text{in}}-\text{RD}{\text{C}}_{\text{out}}\right)∕\left({\text{C}}_{\text{in}}+{\text{C}}_{\text{out}}\right)∕2. \end{array}$$

Where C_in_ is the ^125^I-T_4_ dpm.ml^−1^ per unit volume of entering aCSF; C_out_ is the ^125^I-T_4_ dpm.ml^−1^ of emerging aCSF, F_in_ is the rate of perfusion (μl. min^−1^), and RD is the ratio of Dextran in steady-state of C_in_/C_out_, measured on a Unicam spectrophotometer at 625 nm. The clearance of ^125^I-T_4_ from CSF was corrected for CSF secretion rate (μl. min^−1^), every 10 min throughout each experiment.

#### Brain uptake

At the end of 2 h of V-C perfusion, the regional brain uptake of ^125^I-T_4_ was expressed as follows:

$$\begin{array}{l} {\text{R}}_{\text{Br}}(\text{ml}.{\text{g}}^{-1})=100\times \text{tissue}(\text{dpm}.{\text{g}}^{-1})/(({\text{C}}_{\text{in}}+{\text{C}}_{\text{out}})\\ \;(\text{dpm}.{\text{ml}}^{-1})/2) \end{array}$$

Where C_in_ and C_out_ are ^125^I-T_4_ in inflowing and outflowing CSF from the ventricle, respectively. R_Br_ was then corrected for ^14^C-mannitol content.

#### Statistics

All statistical calculations were performed using Microsoft Excel and GraphPad Prism version 5.0 (GraphPad Inc). Results are expressed as the mean ± SEM. Statistical comparisons were performed using the Student's *t*-test in order to determine statistical significance at *p* < 0.05. Symbols indicate statistical difference: (^*^) *p* < 0.05, (^**^) *p* < 0.001, (^***^) *p* < 0.0001.

## Results

### Percentage recovery and clearance of ^125^I-T_4_ in aCSF after 2 hr V-C perfusion

**Figure 1:**
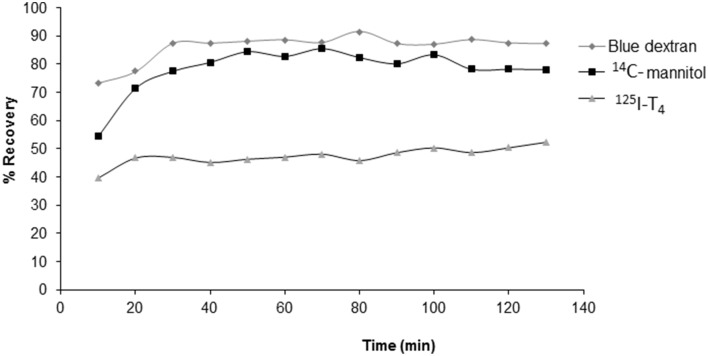
**A representative graph showing the recovery of blue dextran, ^14^C-mannitol and ^125^I-labeled T_4_ in outflow aCSF during 2h of V-C perfusion in rabbits**. Blue dextran was used as an indicator molecule for the newly secreted CSF under experimental conditions, whereas ^14^C-mannitol was used as an extracellular marker. Data are presented as mean ± SEM of 13 samples taken at various time points, *n* = 5 rabbits for each condition.

Results have shown that recovery of blue dextran, used as an indicator dye in this study, and the secretion rate of newly secreted CSF (Figure 1) were consistent with previously published data (Kassem et al., 2007). Indeed, whether or not a drug was added, CSF secretion did not seem to change significantly, after steady state had been reached (*p* > 0.05, Table 1). The mean curves describing the achievement of steady state during V-C perfusion with aCSF containing ^125^I-T_4_ and blue dextran are shown in Figure 1. It's worth noting that ^14^C-mannitol was used as a reference extracellular marker.

**Table 1:** **Recovery and clearance of ^125^I-T_4_ from the aCSF in rabbits, in the presence of various drug treatments**.

	**^125^I-T_4_ Recovery *(%)***	**^125^I-T_4_ Clearance (μl.min^−1^)**
Control	39.87 ± 3.68	43.42 ± 2.52
Brefeldin A (5 μg/ml)	37.10 ± 2.12	46.12 ± 2.41
Low sodium	24.40 ± 2.20^*^	61.65 ± 2.04^*^
BCH (5.0 mM)	27.78 ± 2.20^*^	53.02 ± 1.75^*^
Phloretin (100 μM)	46.98 ± 11.82	41.33 ± 7.86
TA (1.0 mM)	39.23 ± 3.17	44.12 ± 1.83

*Values are given as mean ± SEM*.

* *p < 0.05 (student's t-test), in comparison to control*. *n = 5 for control, n = 3 for each drug*.

The effect of brefeldin A, an intracellular secretory protein blocker, or phloretin, a sugar analog, on recovery and clearance of ^125^I-T_4_ in aCSF did not reach statistical significance (Table 1). However, when low sodium aCSF was used, the recovery of ^125^I-T_4_ in aCSF increased significantly from ~43 to ~62% (*p* < 0.01). This increase in recovery rate was correlated with a significant reduction in clearance by 47% (*p* < 0.001, Table 1), suggesting sodium-dependent uptake of T_4_ from CSF into brain and CP. Moreover, the application of BCH, an amino acid analog, also caused a significant increase in the % recovery of ^125^I-T_4_ when compared to controls (*p* < 0.05, Table 1), which also correlated with a similar reduction in clearance by >50% (Table 1). On the other hand, TA, an OATP inhibitor, did not have any effect on the recovery or clearance of ^125^I-T_4_ in aCSF (Table 1).

### Uptake into CP

**Figure 2:**
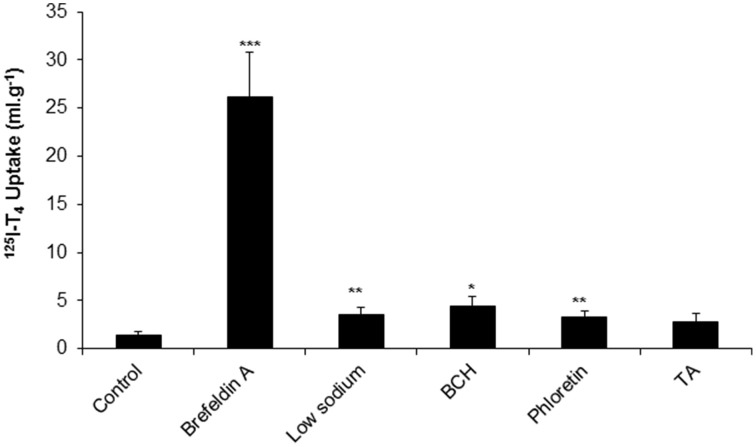
**The uptake of ^125^I-labeled T_4_ into choroid plexus (CP) of rabbits in the presence of brefeldin A, low sodium, BCH, phloretin, and TA**. Data is presented as the mean and error bars represent SEM. Asterisks indicate statistical significance using student's *t*-test between treatment and control for the indicated brain region, ^*^indicates *p* < 0.05, ^**^p < 0.01, ^***^p < 0.001. *n* = 5 for control, *n* = 3 for each drug.

Following the measurement of % recovery and clearance in aCSF, and in order to examine the mechanism of T_4_ transport from CSF, we have explored the uptake of ^125^I-T_4_ into CP. The application of brefeldin A resulted in a significant 18-fold increase of ^125^I-T_4_ uptake in CP compared to controls (~26 ml.g^−1^ vs. 1.4 ml.g^−1^; respectively, *p* < 0.001, Figure 2). Moreover, in the presence of low sodium perfusate, there was a significant 2.5 fold accumulation of ^125^I-T_4_ into CP as compared to controls (~3.5 ml.g^−1^ vs. ~1.4 ml.g^−1^, *p* < 0.01, Figure 2). Similarly, phloretin induced a significant 2.4 fold increase in the uptake of ^125^I-T_4_ into CP (~3.3 ml.g^−1^ vs. ~1.4 ml.g^−1^, *p* < 0.01, Figure 2). Furthermore, BCH significantly enhanced by 3-fold the uptake into CP (*p* < 0.05) compared to controls. On the other hand, TA did not induce any change in ^125^I-T_4_ uptake (Figure 2).

### Uptake into other brain tissues

Since the uptake of ^125^I-T_4_ into CP was significantly raised, we have examined its uptake into specific regions of the brain, namely ER, SER, HC, and CAP. The application of brefeldin A caused more than a 9-fold increase in ^125^I-T_4_ uptake throughout various brain regions such as ER, SER, and the CAP. For example, the uptake into ER and SER was increased by ~10-fold as compared to controls (*p* < 0.05, *p* < 0.001; respectively, Table 2). Moreover, in presence of low sodium aCSF, the highest brain uptake of ^125^I-T_4_ was found into the CAP (Table 2). Brefeldin A treatment also significantly enhanced, but to a lesser extent, the uptake into other brain tissues such as the ER of the frontal cortex and HC (*p* < 0.05, Table 2). It is worth noting that the latter tissues are very close to the ventricles and have more direct surface contact with CSF. This indicates that ^125^I-T_4_ increased uptake into those regions is largely due to a sodium-dependent mechanism. Furthermore, BCH induced the uptake of ^125^I-T_4_ into ER and CAP, which are close to the perfusion site, indicating a role for “L” system in the uptake mechanism. Phloretin treatment also caused a similar increase in the ^125^I-T_4_ uptake into ER and CAP (Table 2), suggesting a phloretin-dependent mechanism as well. Indeed, phloretin treatment represents the second largest effect on ^125^I-T_4_ uptake into brain tissue amongst the drugs tested in this study after that obtained by brefeldin A. Finally, TA treatment resulted in a significant increase in the uptake of ^125^I-T_4_ into the ER and CAP brain regions as compared to controls (Table 2).

**Table 2:** **Effects of various drug treatments on the uptake of ^125^I-T_4_ (ml.g^−1^) in various brain tissues of rabbits**.

**Treatment/condition**	**Uptake of ^125^I-T_4_ (ml.g^−1^) in each brain region**				
	**ER**	**SER**	**CAP**	**HC+ER**	**HC-ER**
Control	0.10±0.03	0.01±0.002	0.1±0.02	0.04±0.01	0.02±0.01
Brefeldin A	1.03±0.10^*^	0.12±0.02^***^	1.0±0.30^**^	0.20±0.04^**^	0.20±0.04^*^
Low sodium	0.30±0.12^**^	0.02±0.01	1.10±0.10^***^	0.10±0.02^*^	0.04±0.01
BCH	0.32±0.20^*^	0.01±0.001	0.21±0.04^*^	0.10±0.02	0.04±0.02
Phloretin	0.40±0.10^**^	0.03±0.01	0.40±0.14^**^	0.10±0.04	0.03±0.01
Taurocholate	0.21±0.04^*^	0.13±0.03	0.20±0.10^*^	0.10±0.02	0.01±0.01

*ER, ependymal region, SER, subependymal region, CAP, caudate putamen, HC-ER, hippocampus with ER being removed. HC+ER = HC plus ER. Values are given as mean ± SEM, n = 3-5*.

* *p < 0.05*,

** *p < 0.01*,

*** *p < 0.001 (student's t-test)*.

## Discussion

Our previous studies have shown that T_4_ transfer from the CSF compartment into lateral CPs and the surrounding brain regions was dependent on TTR and P-gp (Kassem et al., 2006, 2007). The present study reveals that T_4_ transport is affected by the presence of several conditions/drug inhibitors such as brefeldin A, low sodium, BCH, or phloretin and TA. The following lines of evidence support the above statement: (Baehr et al., 2006) the percentage recovery of ^125^I-T_4_, in presence of low sodium or BCH was increased, in comparison to control, compatible with a significant increase in T_4_ clearance, (Goncalves et al., 1990) the uptake of ^125^I-T_4_ into CP was significantly increased in the presence of brefeldin A, low sodium, BCH or phloretin, (Koibuchi, 2013) the uptake of ^125^I-T_4_ into other brain regions, especially ER and CAP, was significantly increased in presence of all drugs as compared to controls, (Francon et al., 1989) TA did not have any effect on the uptake of ^125^I-T_4_ into CP, but enhanced the uptake into ER and CAP.

Consistent with previously published studies, and using the V-C perfusion method, our data revealed that the baseline characteristics of CSF secretion and blue dextran recovery remained stable (Davson and Segal, 1970; Kassem et al., 2007). On the other hand, treatment with brefeldin A, a fungal toxin that disconnects the secretory, recycling, and degradation pathways for membrane proteins (Farwell et al., 1996), resulted into a considerable 18-fold accumulation of ^125^I-T_4_ uptake into the CP. Moreover, it caused a large distribution of ^125^I-T_4_ into the surrounding brain regions such as ER, SER, and HC+ER, when compared to controls. However, brefeldin A treatment had little effect on the % recovery of ^125^I-T_4_. These data suggest that a membrane transport protein system for T_4_ is present at the apical side of the CP tissue and on the CSF side of the ER tissue. Therefore, this transport mechanism is involved in the ^125^I-T_4_ uptake mechanism. Although there was no direct measurement for the effect of brefeldin A treatment on the uptake of T_4_ into CP in the presence of TTR, a role for this protein could also be contributing to this transport mechanism. In fact, previous research has demonstrated that incubation of cultured cells with brefeldin A has prevented active secretion of TTR into the culture medium (Patel et al., 2012).

The transport of ^125^I-T_4_ from CSF to CP and other regions of the brain was demonstrated to be sodium-dependent. Indeed, not only did the low sodium perfusate significantly inhibit the clearance from CSF, but it also increased ^125^I-T_4_ availability in the CSF compartment, and subsequently resulted into a 3 fold accumulation of ^125^I-T_4_ uptake into CPs. Our data complement our previous findings which have demonstrated a sodium-dependent mechanism responsible for T_4_ transport on the apical side of the isolated perfused CP of sheep (Dickson et al., 1987). Previous studies have also shown that CP is an important efflux pathway for T_4_ (Dickson et al., 1987; Kassem et al., 2001), and other compounds (Strazielle and Ghersi-Egea, 1999). The increase in T_4_ uptake in the CP in this study could be due to the inhibitory effect of low sodium on sodium-dependent transporters present at the blood side of the CP. In addition, the low extracellular sodium aCSF may also have an inhibitory effect on Na^+^/H^+^ exchange with blood side of both BBB and B-CSF barriers. Therefore, a reduction in ^125^I-T_4_ efflux across the BBB could subsequently increase its retention within the CSF ventricular compartment. For instance, the increase in ^125^I-T_4_ uptake was also observed in CAP which suggests a similar mechanism. Moreover, in hepatic cells, uptake of TH has been induced by the Na^+^/TA transporting polypeptide (NTCP) and OATP1 (Friesema et al., 1999), which are both found to be expressed at low levels in the rat CP (Choudhuri et al., 2003). Furthermore, the sodium-dependent mechanism of TH has been demonstrated in rat skeletal muscle (Centanni and Robbins, 1987), human glioma cells (Goncalves et al., 1990), rat glial cells (Francon et al., 1989), astrocytes (Beslin et al., 1995), and cerebrocortical neurons (Chantoux et al., 1995). Finally, as a consequence of low sodium effect in this study and results of another study (Kassem et al., 2006), the abundant presence of ^125^I-T_4_in CSF suggests a partial rapid diffusion of ^125^I-T_4_ into tissue that is driven by a bulk flow of CSF and receptor-mediated endocytosis (Kassem et al., 2006).

Following the addition of BCH, an amino acid analog which functions as a substrate for “L” system transporter (Christensen et al., 1989; Stewart et al., 1989), significant reduction in the clearance of ^125^I-T_4_ from CSF was observed. This indicates that ^125^I-T_4_ was inhibited from leaving the perfusate and competed with the “L” amino acid transport system. The high accumulation of ^125^I-T_4_ uptakeinto CPs, following BCH application, is indicative of an inhibition of the “L” system localized at the basolateral side of the CPs (Christensen et al., 1989; Stewart et al., 1989). It is worth noting that another study employing BCH, using isolated perfused CP of the sheep, has reported a slight increase in the ^125^I-T_3_ efflux from CSF to the blood and a significant reduction in the uptake at the blood side (Preston and Segal, 1992). This inhibition of “L” system transport by BCH has been found to contribute significantly to the transport of iodothyronine (T_3_ and T_4_) in a human carcinoma cell line (Powell et al., 2000; Hennemann et al., 2001) and brain astrocytes (Braun et al., 2011). Our data on CPs is consistent with others who demonstrated that “L-type” amino acid transporters (LATs) facilitate TH uptake in xenopus oocytes (Jansen et al., 2005). Furthermore, the transport of T_3_ and T_4_ appeared to be mediated by L and T amino acid TH transporters in mouse neuroblastoma cultured cells (Hennemann et al., 2001). This transport was decreased by BCH, confirming the involvement of the amino acid “L” system (Lakshmanan et al., 1990). On the other hand, ER appeared to have been affected by the reduction in ^125^I-T_4_ clearance from CSF, as a result of “L” system inhibition at the levels of both CPs and BBB. Our data suggests that the “L” system is expressed in ER and CAP and that the T_4_ uptake mechanism into these brain regions studied is BCH-dependent. This is consistent with previous research which indicated that circulating T_4_ preferentially crosses the BBB through several amino acid transporters (Koibuchi, 2013). Our data on BCH in ^125^I-T_4_ transport from CSF into CPs and the brain, across the “L” system, has not been previously reported and may be of physiological importance.

Phloretin treatment resulted in a 3-fold increase in the uptake of ^125^I-T_4_ into CPs, ER and CAP compared to controls. It is well known that this drug has an inhibitory effect on protein binding (McLeese and Eales, 1996), GLUT1 glucose transporter, and the iodothyronine-5′-deiodinase (Movius et al., 1989; Morreale de Escobar et al., 1994). Our data is in agreement with previous studies in the field, which have demonstrated the inhibitory effect of phloretin on the glucose flux from CSF to blood using the isolated perfused CPs of the sheep (Deane and Segal, 1979). It is worth noting that since phloretin binds to the mammalian hexose/sugar transporter (Deane and Segal, 1979), this would involve the energy dependent P-gp. Indeed, the latter has been proven to be involved in the transfer of ^125^I-T_4_ between the CSF, CP, and brain (Kassem et al., 2007). In addition, since there is structural similarities between phloretin and T_3_, more ^125^I-T_4_ can diffuse into the cell by lipid partitioning. In fact, phloretin is known to have an inhibitory effect on glucose transporter (GLUT) in human cancer cells (Wu et al., 2009), and therefore may simply compete with sugar transporters or other unknown T_4_ transporters. Previous studies on rat hepatocarcinoma have reported an inhibitory effect of phloretin on GLUT1 and GLUT2 activity localized on apical and basolateral sides of the CP epithelial cells respectively (Rodríguez-Enríquez et al., 2009; Balmaceda-Aguilera et al., 2012). In addition, it has been proposed that phloretin has competitively inhibited T_3_ uptake into human hepatocarcinoma cultured cells in a dose-dependent manner (Movius et al., 1989). In contrast, phloretin was shown to reduce the ^125^I-T_4_ uptake in isolated trout hepatocytes (Riley and Eales, 1993). Other studies have proven that GLUT1 is localized at the basolateral side of cultured epithelial CP cells (Villalobos et al., 1997) and on both sides of capillary endothelial cells of the BBB (Stewart et al., 1994), hence this transporter may prevent the removal of ^125^I-T_4_ from CSF/brain to the blood. Therefore, it can be hypothesized that the retention of ^125^I-T_4_ by the brain can be the consequence of a decrease in the efflux of ^125^I-T_4_ from the CSF and brain to blood across the brain barriers.

In the presence of TA, the uptake of ^125^I-T_4_ into some brain tissues, such as ER and CAP, was increased. This suggests an interaction between TA and OATP 1a4 since TA was proven to be an OATP 1a4 substrate and sensitive transporter (Do et al., 2013). This agrees with a previous study which demonstrated that OATP2 and OATP3 mediate TH transport when their mRNA is injected into xenopus oocytes (Abe et al., 2002). Furthermore, our data suggests that OATP2 of the abluminal membrane is involved in T_4_ accumulation into those tissues since OATP2 has been identified on the luminal and abluminal membrane of the rat brain capillary endothelial cells (Gao et al., 1999). However, we cannot exclude a role for OATP2 present on the luminal side. Finally, the mediation of T_4_ uptake by organic anion as well as MCT8 was also found in human hepatocarcinoma cells (Ritchie and Taylor, 2010). MCT8, also known as solute carrier (Slc16a2), has been reported to be involved in the uptake mechanism of ^125^I-T_4_ into the brain from the circulation (Kogai et al., 2010; Braun et al., 2011; Horn et al., 2013).

The decrease in TTR synthesis at the blood-CSF-barrier (CP) has been previously shown in aging sheep (Chen et al., 2006). These changes suggested reduced capacity of CP to maintain CSF T_4_ homeostasis, and could also reduce chelation of beta-amyloid, which may result in an added risk for Alzheimer's disease in patients experiencing similar changes in TTR synthesis. In addition, albumin and TTR have been shown to inhibit T_4_ uptake from CSF into isolated sheep brain tissue in a dose-dependent manner (Chen et al., 2005). Therefore, those two proteins (albumin and TTR) prevent the loss of T_4_ from CSF to blood, and enhances the redistribution of T_4_ around the brain.

The increase in T_4_ recovery and clearance in presence of low sodium or BCH, was due respectively to the inhibition of Na^+^/K^+^-ATPase or L-type amino acid transporters. Both of these transporters are localized at the basolateral side of the CP, which was reflected by the accumulation of T_4_ in the CP. The results imply that T_4_ uptake into the CP and brain is largely dependent on the sodium gradient. We suggest that T_4_ transport into the brain and CP is dependent on the concentration of extracellular sodium and/or OATP2 transport mechanisms. Although, treatment with drugs such as Brefeldin A, Phloretin, and TA resulted in a variable increase in T_4_ uptake into CP, they failed to cause a significant change in T_4_ recovery and clearance from CSF. This could be due to either poor penetration of the drugs into the cells, or that the used concentrations were not at optimal levels to produce an effect at the BBB.

In conclusion, the uptake of ^125^I-T_4_ from CSF into CPs suggests the involvement of at least two transport mechanisms among the following: sodium-dependent mechanism, amino acid “L-type” transport system, or OATP transporters. Since the uptake into other brain regions was lower than that obtained in the CPs, we hypothesize that the major efflux pathway of ^125^I-T_4_ from CSF into the brain tissue toward the blood occurs across the blood-CSF barrier, which could exclude any potential role for the BBB in the process.

## Author contributions

NK and KZ conceived the study and designed experiments. NK performed experiments. AE performed statistical analysis. WJ, ME, and SM reviewed data and manuscript. NK and KZ analyzed data and wrote the manuscript. All authors read and approved the final version of the manuscript.

### Conflict of interest statement

The authors declare that the research was conducted in the absence of any commercial or financial relationships that could be construed as a potential conflict of interest.

## Abbreviations

TH: thyroid hormones
T_4_: thyroxine
T_3_: triiodothyronine
CSF: cerebrospinal fluid
CP: choroid plexus
V-C: ventriculo-cisternal
BBB: blood-brain barrier
BCSF-B: blood-cerebrospinal fluid barrier
CNS: central nervous system
BCH: β-2-aminobicyclo- (2,2.1)-heptane-2-carboxylic acid
TA: taurocholate.
